# The impact of colonial-era policies on health workforce regulation in India: lessons for contemporary reform

**DOI:** 10.1186/s12960-021-00640-w

**Published:** 2021-08-18

**Authors:** Veena Sriram, Vikash R. Keshri, Kiran Kumbhar

**Affiliations:** 1 School of Public Policy and Global Affairs and School of Population and Public Health, University of British Columbia, C. K. Choi Building, 251-1855 West Mall, Vancouver, BC V6T 1Z2 Canada; 2 The George Institute for Global Health, New Delhi, India; 3 The George Institute for Global Health, Faculty of Medicine, University of New South Wales, Sydney, Australia; 4 Harvard University, Cambridge, MA USA

**Keywords:** Regulation, Health workforce, Policy, India, Colonialism, Regulatory councils, Historical analysis

## Abstract

**Background:**

Regulation is a critical function in the governance of health workforces. In many countries, regulatory councils for health professionals guide the development and implementation of health workforce policy, but struggle to perform their responsibilities, particularly in low- and middle-income countries (LMICs). Few studies have analyzed the influence of colonialism on modern-day regulatory policy for health workforces in LMICs. Drawing on the example of regulatory policy from India, the goals of this paper is to uncover and highlight the colonial legacies of persistent challenges in medical education and practice within the country, and provide lessons for regulatory policy in India and other LMICs.

**Main body:**

Drawing on peer-reviewed and gray literature, this paper explores the colonial origins of the regulation of medical education and practice in India. We describe three major aspects: (1) Evolution of the structure of the apex regulatory council for doctors—the Medical Council of India (MCI); (2) Reciprocity of medical qualifications between the MCI and the General Medical Council (GMC) in the UK following independence from Britain; (3) Regulatory imbalances between doctors and other cadres, and between biomedicine and Indian systems of medicine.

**Conclusions:**

Challenges in medical education and professional regulation remain a major obstacle to improve the availability, retention and quality of health workers in India and many other LMICs. We conclude that the colonial origins of regulatory policy in India provide critical insight into contemporary debates regarding reform. From a policy perspective, we need to carefully interrogate why our existing policies are framed in particular ways, and consider whether that framing continues to suit our needs in the twenty-first century.

“The General Medical Council takes a special pleasure in this event, since the establishment of the Medical Council of India was in part the result of representations made to the Government of India by the General Medical Council, and many of the provisions of the Indian Medical Council Acts originated in provisions of the Medical Acts of the United Kingdom under which the General Medical Council has functioned from 1858 onwards.”

General Medical Council to Medical Council of India on the latter’s 25th year, 1959 [1]

## Introduction

Regulation is an essential function for ensuring good quality standards of health worker training, availability and performance. In most countries, there is a complex architecture of state and non-state actors governing the health workforce. The effective and transparent regulation of health workforces in many low- and middle-income countries (LMICs) has been a longstanding problem [2, 3]. State-sanctioned, self-regulating bodies, such as regulatory councils, are in particular viewed as mechanisms for professional self-interest and political manipulation, and are burdened with cumbersome bureaucratic structures. As a result, the institutions responsible for health workforce regulation in many LMIC contexts have not yet been successful in ensuring good quality of professional education and training, and have often stood as barriers to health systems strengthening, innovation and reform [3–6].

Left unspoken is how these regulatory systems in many LMICs evolved to their present state. Given the extensive and lasting imprint of colonialism on health systems in these contexts, it is surprising that few have analyzed the colonial origins of health workforce regulation [7–9]. Why are such retrospective, historical analyses needed and what value would these analyses bring to contemporary debates? We argue that such analyses are essential for a comprehensive understanding of the ever-evolving challenges, as without them, we risk repeating the mistakes of the past and limiting our imaginations to a static, narrow set of policy options. Historical analyses surface key learnings regarding why and how certain institutional attitudes and regulatory inefficiencies have persisted and how past experiences can better inform current policymakers on the formulation and implementation of reform.

The experience of India, and in particular, its former regulatory council for doctors, the Medical Council of India (MCI) exemplifies the influence of the colonial legacy in health workforce regulation in LMICs. The regulatory systems institutionalized by the British administration in India set in motion disputes that have been ongoing for over 100 years—disputes regarding the scope of medical practice between doctors and non-physician clinicians on the one hand, and doctors and indigenous medical practitioners on the other; disputes between the federal or union government and the state governments regarding jurisdiction, resource allocation and policy goals; and disputes between the Indian state and foreign governments regarding reciprocity of medical qualifications. In the years following independence, Indian decision makers and medical practitioners have struggled to think beyond this framework introduced in the colonial period, best evident in the persistence of the basic structures and functions of the MCI from its formation in the 1930s to its recent dissolution. Some of the persistent challenges have included an inability to ensure minimal standards of quality in the delivery of training programs at academic medical institutions, widespread allegations of corruption in return for sanctioning private medical colleges, and indistinct governance processes at the national and state levels of medical professional education and licensing [5, 6, 10–13].

Drawing on peer-reviewed and gray literature, this paper explores the colonial origins of the regulation of medical education and practice in India. We describe three major aspects: (1) The evolution of the structure of the apex regulatory council for doctors—the MCI—and the influence of central and state governments; (2) The reciprocity of medical qualifications between the MCI and the General Medical Council (GMC) in the UK following independence from Britain; (3) The regulatory imbalances between doctors and lower level cadres, and between biomedicine and Indian systems of medicine. Through this analysis, our goal is to uncover and highlight the colonial legacies of persistent challenges in practice in India, and thus re-envision regulatory mechanisms for overall health workforce policy in India and other LMICs.

## The evolution of regulatory structures for medical education and practice in India

From 1933 to 2018, the MCI was the apex regulatory body for medical education and medical professionals, before its replacement by a new regulatory body, the National Medical Commission (NMC). This move followed decades of failed reform attempts and was meant to signal a departure from corruption and inefficiency. However, one of the more contentious issues in the formation of the NMC was determining an effective power balance between central and state governments [6, 14].

The Constitution of India mandates division of responsibilities between the union and state government in the federal structure, with some “concurrent” issues being their joint responsibility. For example, the delivery of health services are the responsibilities of the state governments, while the regulation of medical education is a ‘concurrent’ subject [15, 16]. However, the reality of health workforce governance in the country is far more complicated [17, 18]. The funding for public health services is primary responsibility of the state government, with the union government providing additional funding support to states under national health programs. Public medical education is funded by state governments; yet, a small proportion of academic medical institutions located in states are funded by the union government [19]. The regulation of most private and public medical colleges is controlled by the NMC, which is indirectly controlled by the union government, thereby limiting the involvement of states in regulatory functions [6, 20]. Federally funded medical institutions are also given considerable autonomy separate from the NMC through their own legislative Acts [21]. The implications of this fragmentation and lack of cohesion in the governance framework between central and state governments is one of major disconnects between training regulation, health systems and population health needs in India [6].

The seeds for these regulatory debates in India were sown upon the arrival of European health providers alongside the East India Company in the early seventeenth century. As the Company—and later the Crown—expanded its grip on the region, European medical practitioners were organized into provincial medical corps to serve primarily Europeans. Indian subordinates were an essential part of the bureaucracy of “Western”, biomedicine in the subcontinent, given the need for ‘cheap but reliable medical aid for Company servants’ [22]. This led to the formation of cadres of Indian subordinates with some training in biomedicine, such as dressers^1^ and hospital assistants.

The teaching of western medical education in India formally started in 1835 with the establishment of medical colleges in Calcutta and Madras. The expansion of medical colleges and especially medical schools to train subordinates in other parts of British India, was led by provincial governments. By 1938, there were 10 university medical colleges and 27 medical schools, out of which 19 were managed by the provincial government and 9 by non-government organizations [23]. The expansion of medical colleges providing graduate-level qualifications was slower than medical schools awarding licentiate degrees. In the early days, these medical colleges and schools awarded their own diplomas. In 1857, the medical college at Calcutta became affiliated with the local university, with other medical colleges to follow. In the same year, the British government was formally established in the country, bringing with it the initiation of centralized and largely European-staffed administrative and military services, including the Indian Medical Services (IMS) [23–25]. The IMS began to centralize oversight of medical education through its largely European cadre [24]. In 1892, reform in the British Medical Act of 1886 enabled the extension of its jurisdiction leading to the registration of doctors trained in India by the British GMC [24].

Overarching governance reforms in British India in the first two decades of the twentieth century—such as the Morley-Minto and Chelmsford reforms—influenced health policy by bringing health directly under the control of the local provincial government. Medical education on the other hand soon had multiple overseers—the GMC in the UK, the central government in British India through the IMS and central regulatory mechanisms, and the provincial governments.

In 1912, the provinces of India—beginning with Bombay—began constituting their own provincial medical councils (Table 1). These councils excluded indigenous providers—not making them illegal, but making them unregistrable in the ‘official’ system. These Acts also gave the councils considerable power and insulation from provincial governments, including in cases of neglect or abuse of power, while Indian medical graduates continued to be registered and recognized by the GMC [23–25]. The era of centralized oversight appears to have accelerated in the 1920s, when GMC initiated a review of the standard of medical education in India based on reports concerning the teaching of midwifery. In 1928, following some years of periodic inspections through their appointed inspectors, the GMC finally recommended constituting a full-fledged All India Medical Council. The provinces—who on paper still had some level of control—resisted this idea of centralized regulatory institution on the grounds that health was a provincial issue. In 1930, the GMC informed the government that they would derecognize degrees from Indian medical universities [24].

*‘The abandonment of an All-India Medical Council and the non-acceptance by the G.M.C. of an alternative proposal for the appointment as a temporary measure pending the establish of All-India Medical Council, of a board to supervise medical qualification, led the GMC in February 1930 to withdraw the recognition of all the Indian medical degrees. This decision of the GMC completely changed the whole atmosphere and made it imperative to establish a central council’’*

﻿—﻿A. H. Butt﻿,﻿ Secretary of the Medical Council of India in The British Medical Journal, September 14, 1946 [24].

**Table 1:** Path of regulatory institutions in India (1912 - 2020)

	Regulatory Institution			
	Provincial Medical Councils, Various Provinces in British India	Medical Council of India (1933)	Medical Council of India (1956)	National Medical Commission
Legislation/Act	Provincial Medical Act by respective provinces in British India	The Indian Medical Council Act, 1933	The Indian Medical Council Act, 1956	The National Medical Commission Act, 2019
Year of Inception	1912–1936	1934	1956	2020
Statutory Regulatory Body	Provincial Medical Council in most provinces with a Medical College	Medical Council of India (MCI) set up in 1934	Medical Council of India—1956	National Medical Commission (NMC)
Composition of Regulatory Body	President nominated by provincial government, members Elected and Nominated from among medical professionals	President—elected from among members One member from each province—nominated by central government One member from each university with a medical faculty One member from each province with a medical council Four members nominated by central government	President—elected from among members One member from each state nominated by central government One member from each university with medical faculty One member from each province, where a medical register is maintained Seven elected members from among medical professionals Eight members nominated by Central Government	A chairperson appointment by central government 10 ex officio members mostly from medical institution under direct jurisdiction of central government 22 part time members—nominated from among the nominees of state government and state medical councils, university and imminent medical professionals Secretary appointed by the central government
Functions of Regulatory Body	Registration and maintenance of register of medical practitioners Supervision of medical standard and examination Disciplinary control of medical practice Guidance to provincial government on medical education	Maintenance of uniform standard of higher education Oversight of examination Establish reciprocity of medical qualifications Recognition of medical qualification from outside based on reciprocity	Recognition of medical qualification by medical institutions within India and other countries as part of reciprocity Registration of medical practitioners and maintaining Indian Medical Register Maintaining standard of medical course, examination Post-graduate and higher medical education Disciplinary action against registered practitioners	Quality and standard of medical education Regulation of medical institutions, medical research and medical professionals Assess requirement human resource and develop roadmap Professional ethics and promoting ethical conduct Regulating fee of private colleges Other functions as required
Minimum Qualification for Registration and practice	All graduates of medical colleges and Universities in British India/institution outside India as per GMC, UK All licentiate, diploma awarded by different institutions/board in Provinces	All graduates in medicine from Indian university/ institution outside India under reciprocity arrangement Licentiate of Medicine and Surgery only from Bombay, Calcutta and Madras were included	MBBS awarded from recognised institution/university in India and from institution outside India under reciprocity arrangement	MBBS awarded from recognised institution/university in India and Institute/outside India may apply for recognition
Qualification awarding institutions	Medical colleges/universities in British India Medical institution in other selected countries as per Schedule 2 Diploma and certificate by Provincial Government medical schools and other examination boards, councils, or universities	Medical colleges/universities within India Selected universities from outside India	Medical colleges/universities in India Selected universities from outside India	Medical colleges/universities in India Selected Universities from outside India if approved/recognised by the NMC

This led to another round of discussions with provincial representatives, who finally agreed to an All-India Medical Council, despite opposition from the newly formed professional body of Indian doctors, the Indian Medical Association (IMA) [24].

The Indian Medical Council Act, 1933 was thus enacted to constitute a central regulating body of medical education (Table 1). Drawing considerable inspiration from the GMC, the central aim of this act was to bring uniformity in the standard of medical education and examination. There were two key developments in how the center and state now interacted in the context of regulation—(1) the provincial councils retained powers to maintain a register of medical practitioners and disciplinary actions, but lost control over designing province-specific medical education policies; (2) the composition of the Council became a mixture of central and state representatives, and between nominated and elected officials [24]. After the independence, the government of India enacted a new act called the Indian Medical Council Act, 1956 to replace the original from 1933 (Table 1). While the composition of the reformed MCI shared many similarities with its predecessor, there were more central government nominated representatives, as well as elected members of the medical fraternity. Provincial councils were further weakened by shifting final authority to the central level. The ability to start medical colleges came firmly under MCI, thus, curbing provincial government from opening more medical colleges [26].

Experts began seeking cracks in India’s regulatory system even after the new IMC Act, 1956. Various committees from 1961 onwards recommended major changed to the regulation of medical education—by streamlining regulation across the health workforce, maintenance and coordination of high standards in training and expanding the availability of medical colleges [6]. One of the largest regulatory challenges, however, emerged in the 1980s, with the growth of privately run medical colleges, particularly in a few states. In an effort to control the proliferation of low-quality private medical colleges, the IMC Act was amended in 1993 to give MCI further control over regulatory permissions for medical colleges. Unfortunately, it was around this time that MCI was dominated by corrupt factions of elected and nominated members, resulting in the organization becoming embroiled in major corruption scandals around bribes and kickbacks for permitting poor quality institutes [9, 10, 27]. Another emerging issue was the resulting skewed geographic distribution of academic medical institutions and the rapid expansion of private colleges. The MCI and central government could not effectively steward the growth of this expansion leading to unequal distribution of human resources and training institutions [28].

The recent reform in regulatory legislation leading to the formation of the NMC in 2019 is also marred by similar governance challenges observed during the colonial era (Table 1). The NMC in its current form appears to favor centralization of power, further limiting the role of state governments and state councils. Thus, the saga of centralization of the regulatory institution and power dynamic between center-state relationship which started during British colonial rule in India continues to be a major concern even a century later in Independent India.

## Reciprocity with foreign regulatory systems

The functioning of the MCI with respect to one of its major mandates (“maintaining the standard of medical education”) provides another example of how the legacy of colonial-era institutions and attitudes continues to play an important role after independence. As we discussed above, the MCI was established in 1933 primarily as an Indian proxy for the GMC that would perform the job of ensuring that medical colleges in India adhered to GMC-required standards. This helped establish institutional structures, attitudes, and processes in the MCI which privileged, for the purposes of medical curricula and the formal recognition of courses and colleges, the demands of the GMC over demands and requirements of a diverse country, such as India. As Jeffery has argued: “India could have adopted a wide variety of standards of training designed to match varying local needs; or she [sic] might have preferred a single 'national' medical system with the indigenous systems integrated into it. Instead, she [sic] chose a British model.” [8].

Even though the Government of India after independence amended the Indian Medical Council Act, the amendment did not constitute a radical break from the past, and the regulatory orientation towards foreign (mostly British) standards of education remained largely unmodified. One important reason for that seems to have been the continuing relevance of foreign (especially GMC) recognition of Indian medical colleges and degrees. A commentary in the *Journal of the Indian Medical Association* in 1971 argued that there had not been much “Indianisation of medical education, because most of our senior leaders in medical education today have studied abroad and have tried to develop, very faithfully indeed, our medical education along the lines which existed in those foreign countries.” [29] It was alleged that these leaders had failed to follow the social developments in India, insisting on adhering to the standards they saw in foreign nations [9] and thereby creating a mismatch between regulatory standards, such as curricula, distribution of colleges and postgraduate programs, and the needs of people and communities in different parts of India.

The derecognition of Indian medical degrees by the General Medical Council in May 1975 brought this foreign orientation of the regulation of Indian medical education in sharp relief. The statement of the GMC acknowledged that the “medical curriculum in India is still based on the British pattern,” but expressed that the growing number of medical colleges in India, among other factors, had made it impossible for the GMC to “properly” exercise their function of “safeguarding by registration the public of this country [the United Kingdom]” [30].^2^ There were two broad types of reaction in India to the derecognition by GMC: one of disbelief and anger, and the other of resigned helplessness at what were considered to be “declining” standards in India [31]. But as Jeffery has argued, neither of these reactions questioned the basic orientation of Indian medical education towards the pattern and standards that were put in place during the colonial period, almost exclusively to satisfy the demands of a foreign medical council [8].

In fact even after the GMC derecognition, the Indian state continued its adherence to foreign standards: there was no radical overhaul in how the MCI approached medical education, and the union government soon established a new degree-granting body called the “National Board of Examinations” (NBE) to award “prestigious” postgraduate degrees [32]. The NBE was formed in 1975, initially a part of the National Academy of Medical Sciences, until 1982, when it became an autonomous body under the Ministry of Health and Family Welfare [33]. The government claimed that NBE would "ensure inter alia… availability of prestigious qualifications within the country comparable to similar qualifications given in the foreign countries and thus minimise the tendency [of doctors] to go abroad."[34] A contemporary commentary expressed skepticism about the NBE leading to any such changes [35].^3^ In 1977, Jeffery observed that the Indian government was still “committed to the model of specialisation and standards of the West.” [8]. Today, India is one of few, if not the only, countries with two parallel post-graduate medical education regulatory bodies. The implication of this parallel system is unstandardized curricula for postgraduate training, decades-long litigation between the two organizations regarding degree equivalence, and a lack of cohesiveness in the policy frameworks guiding postgraduate education to the detriment of effective workforce planning [5, 10, 36].

In 1981 the Indian Council of Medical Research and the Indian Council of Social Science Research released a report titled “Health for All: An Alternative Strategy.” This was meant to be an “alternative” to the “urban-biased, top-down, and elite-oriented approach of the British period” which, according to the authors, still continued to dominate health services in the country [37]. This report seems to have inspired the 1982 National Health Policy (NHP), which declared that the “existing situation has been largely engendered by the almost wholesale adoption of health manpower development policies and the establishment of curative centres based on the Western models.” [38]. However, three years after the NHP was declared, public health physician Dr Abhay Bang wrote that “medical education today runs totally against” the NHP [39]. Dr. Bang reiterated that India needed “community physicians trained to run primary health centers effectively. This will need drastic changes in the medical curriculum and culture. The Western norms of medical education followed by the Medical Council of India need to be thrown away.” [39]. Such drastic changes have not yet taken shape in India, and even today, medical education struggles to shake off the metropolitan, urban orientation of curriculum and is failing to make graduating physicians more attuned to the needs and requirements of the local communities and primary health centers.

At the same time, a more contemporary form of the reciprocity debate is taking place in recent years with thousands of Indian medical students attending medical school in China, Russia, Ukraine and other countries, returning to India and struggling to pass an entrance examination, the Foreign Medical Graduates Examination, coincidentally organized by the NBE [40]. This exam has a notoriously low rate of passing, with only 14.2% of exam takers passing between 2015 and 2019 [40]. Thousands of doctors trained in these countries are currently living in India, unable to practice despite an ongoing shortage of trained medical providers in many parts of the country [41].

## Regulatory imbalances between biomedical doctors and LMPs, and biomedicine and Indian systems of medicine

There were two key repercussions of the early regulation of biomedicine in India. The first is that by primarily focusing on the production of MBBS doctors without wider systemic changes to facilitate the relocation of these doctors from urban to rural areas, the British, and later the Indian government, began a maldistribution of health workers that continues to this day. The two potential policy levers to address this maldistribution—non-physician clinicians and providers of indigenous medicine—have been fraught with regulatory challenges for two centuries.

As noted earlier, Indian subordinates were an essential part of the growth of modern medicine from the East India Company’s early days. As the Company formalized its rule, this led to the formation of official cadres of Indian subordinates with some training in Western medicine, such as dressers and hospital assistants. Starting in 1911, the graduates of training programs for subordinates were called sub-assistant surgeons, and from 1912, they could have the term ‘Licensed Medical Practitioners’ or LMP attached to their names [7].

LMPs made up a vast majority of practitioners in the country by the twentieth century. By the late 1930s, LMPs made up 30,000 of the approximately 40,000 allopathic medical providers in India. LMPs played a crucial role in rural health schemes, serving in parts of the country, where graduate doctors were unwilling to relocate [7]. However, despite being promoted as a ‘cheap’ form of labor, the cadre operated under a nebulous regulatory environment.^4^ As noted under the Bhore Committee report, “the absence of a central body to control medical school education has naturally led to a wide divergence in standards in the training given in the different schools and, of late, owing to the growing demand of for doctors an increasing number of students has been admitted every year to the already congested, ill equipped and understaffed schools.” [42].

The issue that ultimately sealed the fate of LMPs, however, was the oversight of the cadre by medical graduates through regulatory councils, and in particular, the regulation of their registration. As noted above, between 1912 and 1919, all provinces passed medical registration acts, and had discretion in terms of the types of medical practitioners that would come under their purview [43]. The Madras Medical Registration Act of 1914 recognized LMPs as qualified medical practitioners, and sought to bring these practitioners under “disciplinary control”. However, the Indian Medical Council Act in 1933, marked “the beginning of the final phase of life for licentiates” [7], and excluded LMPs from the register, essentially making them ‘unqualified’ in the eyes of the regulatory system. Roger Jeffery argues that this was done primarily so that the new Indian Medical Council (later MCI) could be able to continue to maintain the recognition offered to Indian medical qualifications by UK's GMC. Government administrators in India feared that including licentiate qualifications in the All India Medical Register could prove counterproductive to gaining the confidence of the GMC which already had been raising questions about the quality of medical education in India [25]. The Government of India in 1938 reviewed licentiate education, and in 1942, the Indian Medical Council announced that medical schools should be abolished or converted to medical colleges [42].

Ironically, the decision had immense repercussions for the availability and equitable distribution of health services in India. The Indian medical profession has struggled for decades with the issue of rural availability of doctors. ‘Unqualified’ allopathic providers—sometimes known as Rural Medical Practitioners continue to practice widely [44]. From a regulatory standpoint, the qualified medical professionals have been highly resistant to RMPs, fighting on multiple occasions to limit opportunities to train these through abbreviated training courses. In the latest iteration of this debate, the Government of India and the NMC announced creation a new non-physician cadre of Community Health Providers (CHP), with unclear regulatory implications. Details regarding the training and career progression of the CHP cadre has not been clarified at the time of writing [45].

Traditional practitioners of medicine were at the mercy of the ‘newly’ imposed system of modern medicine that arrived with colonialism. Despite some early efforts to understand the value of traditional medicine and some efforts at exchange between the systems, “British criticisms of indigenous medicine became increasingly strident and intolerant” [43]. For example, the Native Medical Institution was set up in Calcutta in 1822 to inexpensively train ‘native doctors’ for the East India Company, allowing for some integration between allopathic and traditional systems of medicine as a way to recruit trainees and slowly inculcate an appreciation for Western medicine. However, from 1835 onwards, due to the acceptance of Macualey’s Minute on Education and its premise that education in India should be anglicized, the focus of the institution, and the two others like it, shifted entirely to allopathic medicine. Traditional practitioners were not excluded from the public health system—in fact, hakims were employed in Punjab as vaccinators and health extension workers [46]. Eventually, the enterprise of training these practitioners became a private, unregulated system.

Another regulatory challenge was the formal registration of traditional medicine providers. The registration acts that appeared between 1912 and 1919 excluded traditional medicine providers. The representatives of these providers noted presciently that this type of regulation is incompatible with the diversity of systems of medicine in India, stating that this act "may be justified in countries, where only one system of medicine is pursued, not in India, where the masses depend on [different systems] of medicine" [7]. The result of these decisions was the regulatory bifurcation of medicine in India, which never truly reconciled. Eventually, the Central Council of Indian Medicine was established in 1971, to oversee education in various systems of Indian medicine [47], followed more recently by an independent Ministry of Ayurveda, Yoga, Unani, Siddha and Homeopathy [48]. Indian medicine continues to be at odds, particularly around policies and proposals that would provide traditional medicine providers with official sanction to engage in providing biomedicine in limited manner. This debate has taken on particular urgency in recent years with the co-location of traditional and biomedical providers in public sector health facilities, and due to the fact that traditional medicine providers often the only clinical staff in public sector facilities in more rural, remote locations due to challenges in recruiting biomedical providers to work in these areas.

## Conclusions

Challenges in medical education and professional regulation remain a major obstacle to improving the availability, retention and quality of health workers in many LMICs. In this paper, we argue that an understanding of the colonial origins of regulatory policy in India provide critical insight into the challenges observed today. The persistence of British colonial-era structures and institutions has been a theme in many sectors in India, including legislative, judiciary, and criminal justice apparatuses [49]. Other health professions in India, such as nursing, have also faced enormous challenges, in part due to regulatory systems established during colonial times or shortly after [50]. These institutions are also in the process of reform [51]. Seen in this broader context, the continuing colonial legacy in healthcare, and more particularly in the regulation of medical education and training as described above, does not come as a surprise. However, the literature on health workforce regulation in LMICs rarely takes into account these historical origins, and as a result, often misses the key underlying reasons for continued inefficiencies and bottlenecks.

From a policy perspective, we need to carefully interrogate why our existing policies are framed in particular ways, and consider whether that framing continues to suit our needs in the twenty-first century. As India has grown to a country of over 1.3 billion people with 36 states and territories, does the British-era policy of centralizing the regulation of medical education continue to hold? Can we revisit why the country started the NBE in the first place, and assess whether it is pertinent to have what is likely the only parallel system of postgraduate medical education in the world? Can we learn lessons around why systems of medicine were kept distinct and put these regulatory systems in conversation with one another, rather than in opposition? There are rich lessons to be learnt for future health policy in LMICs by looking into the past—and being ready and willing to evolve new regulatory institutions that meet our current moment.

## Data Availability

Not applicable.

## Abbreviations

GMC: General Medical Council
LMICs: Low- and middle-income countries
LMP: Licensed medical practitioner
MCI: Medical Council of India
NMC: National Medical Commission

## Footnotes

1 Cadre of hospital assistants with responsibilities including bandaging, stitching, injections, dressing of wounds and other tasks to assist surgeons and medical staff—https://www.nqr.gov.in/sites/default/files/QF%20_Dresser%20_MED206.pdf

2 Indian medical gradates currently seeking to practice in the UK must undertake entrance examinations or complete training programs recognized by regulatory bodies in the UK—https://www.gmc-uk.org/registration-and-licensing/join-the-register/before-you-apply/working-as-a-doctor-in-the-uk

3 In 2008 the Ministry of Health and Family Welfare amended the Indian Medical Council Act to recognize post-graduate medical qualifications from five foreign countries: Australia, Canada, New Zealand, U.K. and U.S.A. Notification accessed on 12 July 2021: https://main.mohfw.gov.in/medicaleducationcounselling/recognition-foreign-post-graduate-medical-qualification.".

4 "It is worth noting that the Medical Council of India was concerned with the regulation of standards only in the university-affiliated medical colleges, while the medical schools which granted the licentiates were under the control of provincial governments.".
